# The superiority of graphics over text in long-term memory retention

**DOI:** 10.3758/s13423-025-02708-3

**Published:** 2025-05-08

**Authors:** Lorenzo Ciccione, Denis Caroti, Syalie Liu, Valeria Giardino, Elena Pasquinelli, Stanislas Dehaene

**Affiliations:** 1 Cognitive Neuroimaging Unit, CEA, INSERM, Université Paris-Saclay, NeuroSpin Center, 91191 Gif/Yvette, France; 2 Department of Psychology, Université Paris 8, DysCo Lab, 93526 Saint-Denis, France; 3 Centre Gilles Gaston Granger, UMR 7304, Aix Marseille Université, CNRS, Aix-en-Provence, France; 4 Centre Maurice Halbwachs, UMR 8097 ENS- EHESS- CNRS- INRAE, 48 Boulevard Jourdan, 75014 Paris, France; 5 Collège de France, Université Paris Sciences Lettres (PSL), 11 Place Marcelin Berthelot, 75005 Paris, France; 6 Institut Jean Nicod, CNRS, 29 Rue d’Ulm, 75005 Paris, France; 7 Fondation La Main À La Pâte, 75006 43 Rue de Rennes, Paris, France

**Keywords:** Graphical representations, Data visualization, Memory, Learning, Educational tools

## Abstract

Graphical representations of data are pervasive in modern communication and are often used to convey socio-economic, scientific, and medical information. Despite their popularity, it is still unknown whether they can enhance the long-term retention of their content. We conducted a delayed-recall task with psychology undergraduates (N = 92), in which participants read about the evolution of a socio-economic phenomenon, with five to six datapoints presented as graphics, text, or table; recall was operationalized as correct reporting of the trend in the data, 2 h after the information was presented. We found that graphics facilitated the delayed recall of such trends. No advantage was found on immediate recall of trends or specific datapoints in another sample of participants (N = 80). Thus, even for equal initial encoding of data, and even for very concise materials, graphics facilitate long-term retention. Overall, the study reveals the potential of graphics as effective tools for enhancing memory retention and therefore highlights their valuable role in educational settings.

## Introduction

Graphical representations of data have been used for decades, both as a tool for socio-economic and scientific communication (Archambault et al., 2015) and as a means of statistical data exploration (Anscombe, 1973). From early maps to astronomical diagrams, progressive innovations in measurement and theory have continuously redefined the field of data visualization, in which graphs not only depict information, but also reveal phenomena that would be much harder to inspect using text or mathematical formulas (Friendly, 2008). With the advent of information technologies, data graphics have become increasingly prominent in everyday life, and the ability to interpret them, otherwise known as “graphicacy” (Balchin & Coleman, 1966) or “visual literacy,” is considered an essential skill for the third millennium. Understanding graphics is not a particularly developed skill in the contemporary world (Galesic & Garcia-Retamero, 2011), and this seems particularly true for complex graphics (Locoro et al., 2021); also, visual literacy is highly variable in the general population (Pandey & Ottley, 2023). However, graphs are frequently employed at school for both teaching and assessment (Lowrie & Diezmann, 2009), and using them is considered indispensable for any journalist, economist, or scientist.

It is unclear whether graphics are truly effective in facilitating data memorization, or if their increasing use is related to fashion, esthetics, or even a decreasing willingness to engage in intensive reading. Psychological experiments generally support the efficiency of pictorial representations (“A picture is worth a thousand words”). For instance, visualizing an image corresponding to written text facilitates the understanding and memorizing of its content (Clark & Paivio, 1991; Paivio & Csapo, 1973) and, more generally, pictures are better remembered than words, a phenomenon referred to as the “picture superiority effect” (e.g., Standing et al., 1970; Stenberg, 2006). When pictorial information is added to text, it can improve students’ learning (Carney & Levin, 2001), especially if the visual is in line with readers’ preferences (Walsh et al., 2021), and students report being helped in their learning by the presence of graphs in textbooks (Jamal & Mustaffa, 2023). The mere presence of a visual hypertext increases the probability of remembering the content of the text in which it appears (Lin, 2004). The question of whether there could be a “graph superiority effect,” however, remains open. In theory, a single graph can indeed summarize, in a concise and effective way, information that would be much more extensive if presented in textual or tabular form – but such conciseness is not always beneficial: it could be detrimental to the depth of processing, which is a well-known determinant of memory (Brown et al., 2014). Theoretical analysis suggests that graphics owe some of their usefulness to the fact that they make explicit, in spatial format, some of the algebraic relations among the plotted data, thus making them available to efficient and often parallel visual search and recognition processes (Larkin & Simon, 1987). In agreement with this idea, studies have found that graphics are better than tables in tasks where participants have to detect or compare trends (Jarvenpaa & Dickson, 1988; Washburne, 1927), to predict future events based on data series (Umanath & Vessey, 1994) and, more generally, when data are not random and follow a recognizable trend (Meyer et al., 1999). Pie charts and line charts also seem to be superior to tables in perceptual tasks involving the simple comparison of values (Spence & Lewandowsky, 1991), whereas tabular representations help locate precise values (Lalomia & Coovert, 1987; Meyer, 2000; Meyer et al., 1997). Recent studies have shown that humans, regardless of education and age, possess intuitive abilities to extract the linear trend of a scatterplot (Ciccione & Dehaene, 2021; Ciccione et al., 2023) or to predict the evolution of an exponential curve (Ciccione et al., 2022).

Beyond the extraction of trends and patterns, an additional advantage of graphics has also been observed in decision making, although not always replicated (Van Der Meulen et al., 2010). For example, extracting clusters of datapoints from graphics helps readers reason about the content of the plot (Ratwani et al., 2008); financial experts are significantly helped by the presence of graphics when asked to make decisions based on numerical information (Cardoso et al., 2016); and graphics improve medical decision making and Bayesian reasoning (Ottley et al., 2016). Adding graphical representations to textual paragraphs has also many other advantages: it increases the likelihood of remembering the general topic covered by the plot (Borgo et al., 2012) and its title (Peña et al., 2020), and also improves problem-solving skills and understanding of the phenomena discussed (Mayer, 1999).

Much research has also been dedicated to the ergonomics of graphics design (Franconeri et al., 2021; Kosslyn, 2006), but much less effort has been devoted to the subsequent memory processes. Some of the factors that make data visualizations more memorable have been identified (Borkin et al., 2013), and it has been proven that well-executed graphics are more easily remembered (Borkin et al., 2016) and that embellished plots help to remember the trend of the data compared to minimalist ones (Bateman et al., 2010). However, a distinction should be made between the memorability of graphics and that of the content they communicate. When it comes to long-term memory of the *contents* of the graphics, little data seems available, besides some evidence suggesting that, when asked to explicitly memorize information for an immediate recall test, participants presented with graphics are better at reproducing the ranking in the data and at detecting patterns (Schmell & Umanath, 1988).

The aim of the current study was to establish whether graphical presentation of trend information was associated with superior delayed recall of that trend, in comparison with tabular or textual presentation. To address this issue, 2 h after observing five or six data points presented in graphic or text form, we asked participants, without forewarning, to answer verbal questions about the evolution of the data. Three aspects of the design are worth stressing. First, we used very small datasets, so that they could reasonably be apprehended from a table or a short text; indeed, the present experiment can be seen as a new test of Tufte’s (2001) suggestion that, for such a concise dataset, a visual plot is unnecessary (for evidence to the contrary, see Carswell & Ramzy, 1997). Second, we tested whether, in immediate recall, a separate group of participants performed well, independently of input format, thus evaluating the quality of initial data encoding. We reasoned that the identification and comprehension of a small amount of numerical information may not be affected by the mode of presentation, but its memorization might. Third, the answer to the questions we asked could literally be found in the text form of data presentation. Thus, we predicted that on immediate recall, the text condition would be as easy as graphics, whereas the superiority of graphics would only emerge in the longer term, i.e., in the subsequent recall of the information. In addition to those two critical conditions (graphics vs. text), we also tested two additional exploratory conditions: a data table which, for such small data sets, was also expected to yield good immediate access to information; and a misleading graphic condition in which the scale was inappropriate given the data trend, and which was therefore expected to yield high error rates – this condition was included to verify the sensitivity of our measures.

To anticipate the results, graphical displays were indeed superior to text in delayed retention. Thus, data graphics, with their highly readable syntax of axes and variables, constitute a highly effective format with which to summarize data, allowing it to be efficiently stored and retrieved from memory.

## Methods

### Experiment 1: Delayed recall

#### Participants

Ninety-two psychology undergraduate students (81 women, 11 men; age: 21.6 **±** 6.8 years) took part in a delayed memory task, on a voluntary basis. They all received basic statistical training as part of their degree. A power analysis based on G*Power (Faul et al., 2007) determined, for our main ANOVA on error rate (parameters used: four groups, four measures per group, α = 0.05, power = 0.9, an expected moderate-to-large effect size of 0.35 and an expected correlation of 0.5 among repeated measures), a sample size of 80 participants.

#### Materials

**Fig. 1:**
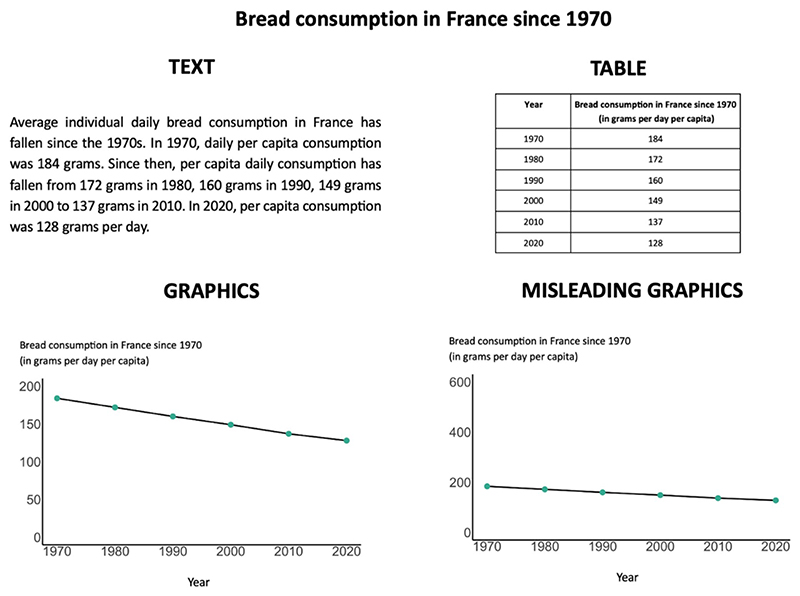
Representation of information for the four experimental groups. In both Experiment 1 and Experiment 2, the same quantitative information (in this case, the bread consumption in France since 1970) was presented in one out of four different formats, depending on the group to which participants were randomly assigned to: as a text, as a table, as a graphics, as a misleading graphics. Note that the labels in the figure have been translated into English for the purpose of this article: all materials were presented in French to participants

Unbeknownst to participants, they were randomly divided into four groups (according to a between-subjects design), corresponding to the four ways in which the same socio-economic topics were displayed in a booklet: all as graphics, all as tables, all as text paragraphs, or all as misleading graphics (in the latter, the y axis was zoomed in or out in order to reduce or amplify the visual trends; this condition was added to ensure that our behavioral measures were sensitive enough). Figure 1 shows an example topic presented in the four modalities. Topic 1 described an increasing trend with six data points; topic 2 described a decreasing trend with six data points; topic 3 described a stagnating trend with six datapoints; topic 4 described numeric values for five levels of a categorical variable. For the graphics’ and misleading graphics’ condition, topics 1, 2, and 3 were represented with a line graph, and topic 4 with a bar plot.

#### Procedure

The experiment was conducted in the context of a second-year cognitive psychology class. First, after providing signed written consent, participants were randomly given one of the four booklets described above (containing either texts, tables, graphics, or misleading graphics) and they were asked to keep it closed and to simply note an identification code written at the top. Then, they were informed they would read about four socio-economic topics (presented in a random order) and that they had 40 s to read each topic, one per page (the timing was given by the experimenter: after 40 s, students had to turn the page and read about the new topic). They were explicitly asked to stay focused for the given amount of time; no other instruction was given to them. After having completed this first task, participants were asked not to talk about the experiment with their colleagues. A 2-h class about emotions and reasoning (thus unrelated to the experiment) was administered. After the class, without forewarning, participants were given new booklets containing the test (which was identical for all groups), on which they added the identification code noted before (in order to allow the experimenter to pair each subject with the corresponding group). Recall for each topic was demonstrated in this phase by correctly responding to four questions about the four topics. For topics 1, 2, and 3, the four response options had the following structure: the described phenomenon increased; it decreased; it stagnated; it had a peak. For topic 4, the options were: India has a gold consumption three times higher than other regions; six times higher; fairly comparable; three times smaller. Crucially, the response options were presented with the exact same sentences used in the text condition. Thus, only the participants in the text condition had actually read the same information, using the same formulation as in the subsequent questions. After responding to these four questions, participants were invited to indicate their age and gender and, lastly, to take a short graphicacy scale (Okan et al., 2019), which, for the sake of participants’ understanding, was translated in French. This scale consisted of four multiple-choice questions about graphical representations and aimed to quickly evaluate basic graphicacy skills. The whole testing phase had to be completed in 10 min at most.

#### Analysis

For each participant, we computed the average error rate over the four questions about the socio-economic topics they previously read about (chance level is 75% errors). Several ANOVAs on the dependent variable (error rates) were performed. Graphicacy skills were evaluated as the number of correct answers (ranging from 0 to 4) in the short graphicacy test. For simplicity, performance ranging from 0 to 2 was coded as “low graph literacy,” and 3 to 4 as “high graph literacy.”

### Experiment 2: Immediate recall

#### Participants

Eighty psychology undergraduate students (73 women, 7 men; age: 21 **±** 5.2 years) took part in an immediate-recall task, on a voluntary basis. They all received basic statistical training as part of their degree. The sample size was determined using the same power analysis described for Experiment 1.

#### Materials

The same booklets described in Experiment 1 were presented to participants.

#### Procedure

The experiment was conducted in the context of a second-year cognitive psychology class. Exactly as for Experiment 1, after giving written consent, participants were given one of the four paper booklets and were allowed to examine the data about each topic for 40 s. The difference was that, after this period, students had to turn the page and answer three questions, in less than 1 min, about the topic they had just read about (without the possibility to re-read it). The first question was the same as in Experiment 1 (it asked about the trend of the evolution presented); the second question asked about the numerical range of the data; and the third asked about a specific numerical value. For topic 4, both the second and third questions asked about specific values. After 1 min, participants were invited to turn the page and to read about a new topic. After all topics (and relative questions) were presented, participants had to indicate their age and gender and to complete the short graphicacy scale.

#### Analysis

The performance for question 1 was evaluated exactly as for Experiment 1. To evaluate the performance for questions 2 and 3, we first calculated the percent deviation from the correct answer (for each subject) for each topic (i.e., the absolute difference from the expected correct value and the given value, divided by the expected value, multiplied by 100). Then we computed the median percent deviation over the topics. Omitted responses (3.6% of the total answers) or percent deviations higher than 100 (8% of the total answers) were capped at 100. Several ANOVAs on the dependent variables (error rates and median percent deviations) were performed. Graphicacy skills were evaluated as for Experiment 1.

## Results

### Presenting information as a graphics improves delayed recall

**Fig. 2:**
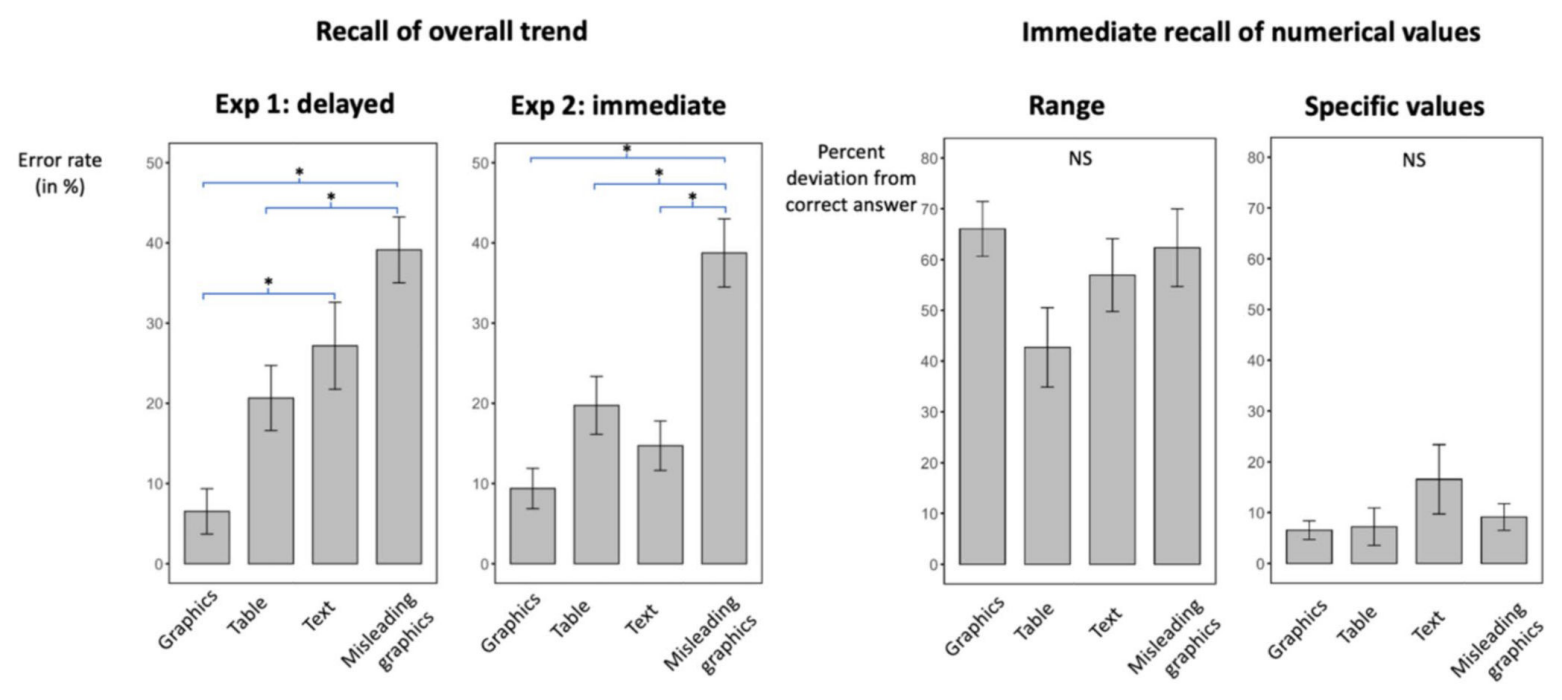
Performance depending on the experimental condition. **A**. Error rate (in percentage) for the question about the overall trend of the data, plotted as a function of the experimental condition to which each subject was randomly assigned to, in Experiment 1 (delayed recall; first plot) and in Experiment 2 (immediate recall, second plot). **B**. Percent deviation from correct answer in Experiment 2 (immediate recall of numerical values): left, question on the numerical range of data; right, question about specific values. The asterisk (*) indicates a significant difference (*p* < 0.05) between conditions. Data are plotted as means ± SEM

Participants in Experiment 1 were asked about the general trend of data they had read about two hours before. Figure 2 (first plot) shows the error rate (in percentage) across topics and subjects for each experimental group. The error rate varied significantly across the four presentation formats (ANOVA: F[3, 88] = 10.5, partial η^2^ = 0.26, *p* < 0.001). Post hoc Tukey tests indicated that the lowest error rate, which occurred in the “graphics” condition (6% errors), was significantly smaller than the mean error rate for the “text” condition (27%, *p* < 0.01). Furthermore, the other two conditions also led to greater error rates than graphics: “misleading graphics” condition, 39% errors, *p* < 0.001; “table” condition, 21%, *p* = 0.05.

### No advantage of graphics in immediate recall

Participants in Experiment 2 were asked three questions about each topic, immediately after they read it. The first question was the same as in Experiment 1 and pertained the general trend of the data. The error rate varied as a function of the input format (ANOVA: F[3, 76] = 14.9, partial η^2^ = 0.37, *p* < 0.001) but a post hoc Tukey test revealed that only the error rate for the misleading graphic condition differed significantly from any of the others (all corresponding *p* values < 0.01) (see Fig. 2). In particular, there was no difference between the two crucial formats, graphics (9.4% errors) and text (14.7% errors; *p* = 0.43). When we ran a 2 × 2 ANOVA on all subjects restricted to these two input formats (with input format and condition – immediate or delayed – as between-subjects factors), we found a main effect of the input format (F[1, 83] = 11.96, partial η^2^ = 0.13, *p* < 0.001), and an interaction of input format and condition (F[1, 83] = 4.16, partial η^2^ = 0.05, *p* < 0.05), indicating that the advantage for graphics over text was significantly larger in delayed than in immediate testing.

Concerning the two questions that were only tested in immediate recall, i.e., those about numerical range (for topics 1, 2, and 3; third plot) and specific values (topics 1, 2, 3, and 4; fourth plot), no effect of the mode of presentation on percent deviation from correct was found (numerical range ANOVA: F[3, 76] = 2.2, partial η^2^ = 0.08, *p* = 0.1; specific values ANOVA: F[3, 76] = 1.3, partial η^2^ = 0.05, *p* = 0.3), nor were specific differences found in post hoc Tukey tests (all *p* values > 0.05). We found analogous results when, instead of the percent deviation, we computed the ratio between the correct value and the expected one (both ANO-VAs led to p values > 0.22).

### Graphicacy was a significant predictor of immediate but not delayed recall

As explained in the *Methods*, we defined participants scoring 3 or more in the graphicacy scale test as having high graphicacy, and those having obtained a score of 2 or less as having low graphicacy (the average graphicacy score, over both experiments, was 2.44 out of 4). We then repeated the ANOVA on error rates from experiment 1, now with input format and graphicacy as between-subjects factors. The effect of input format remained significant (F[3, 84] = 10.13, partial η^2^ = 0.27, *p* < 0.001) but neither the main effect of graphicacy nor the interaction reached significance (both *p* values > 0.54). For Experiment 2 (question 1), the same ANOVA revealed a significant effect of input format (F[3, 72] = 15.2, partial η^2^ = 0.39, *p* < 0.001) and a significant effect of graphicacy (F[1, 72] = 5.07, partial η^2^ = 0.07, *p* < 0.05; with participants having a high graph literacy performing overall slightly better than those with a low graph literacy), but no interaction between the two factors (*p* = 0.44). For the second question of Experiment 2 (the one about numerical range), an ANOVA on the percent deviation found a significant effect of the input format (F[3, 72] = 3.48, partial η^2^ = 0.13, *p* < 0.05), but not of graphicacy (either alone or in isolation; both *p* values > 0.13). For the third question of Experiment 2 (the one about specific values), no effect of input format was found (*p* = 0.41) and the graph literacy had no effect in isolation (*p* = 0.37) but it entered into a significant interaction with the input format (F[3, 72] = 3.25, partial η^2^ = 0.12, *p* < 0.05).

## Discussion

Graphics have long been recognized for their ability to concisely and effectively convey complex quantitative information. This study aimed to explore whether the advantages of graphical representations extend to enhancing long-term memory retention of the content they convey. Specifically, we investigated if information presented as graphics is more likely to be remembered compared to text and tables.

In a delayed-recall task, those who had initially viewed information as a graphics demonstrated significantly higher accuracy in recalling the general trends of data after a 2-h delay, suggesting that graphics improve long-term memory retention. This finding held independently of the graphicacy level of the participant. The ability of graphics to visually depict trends and patterns likely provides cognitive anchors that help retain the overall behavior of the data (Ratwani et al., 2008), making it easier to recall if compared with tables and text, for which the appearance of a trend in the dataset is less obvious (Vessey, 1991).

Importantly, participants in the text condition read the exact same sentence that was later presented as an answer option in the test. And yet, they were not the ones showing the best performance.

Importantly, in a separate sample of participants performing an immediate-recall task, there was no significant advantage of graphics over text and tables in the recall of the overall trends. Furthermore, when comparing immediate versus delayed recall of graphics versus text, a significant interaction of delay length and input format confirmed that graphics, relative to text, significantly facilitated the retention of information. There was also no advantage of graphics in the immediate recall of the numerical range and specific values. These findings are important, since they suggest that the advantage in the delayed-recall task is unlikely to be due to a better encoding of the numerical information during the reading phase. In the present experiment, we did not expect a graphical advantage in the immediate recall, since the material was small and concise: in other words, whether participants saw graphics, a table, or a text should not (and did not) make any difference to their ability to extract a range or a specific value when tested just a few seconds after the material’s presentation. The real advantage of graphics seems to concern the maintenance of the information in memory, even under conditions where its immediate understanding is not significantly better than that for text. It is important to note, however, that the absence of a graphical advantage in the immediate recall of information cannot be taken as a true null effect, especially given the size of our samples. Future studies should investigate whether such a null effect continues to hold in a larger sample of participants.

Crucially, we presented as stimuli only datasets with a small number of datapoints, precisely in order to avoid a clear advantage of the graphical modality. Richer datasets would likely show an even greater advantage, as their trend would still be readable at a glance in the graphical format but would likely be too difficult to extract in the same amount of time from a table or a text. Graphicacy expert Edward Tufte proposed that, for small datasets, a table is better than graphics (Tufte, 2001). While this might be true for the immediate encoding of information (although no such evidence was found in our study), this is clearly not beneficial if the purpose is making a trend more memorable in the long-term.

The findings of this study could have profound implications for educational practices, at least for those readers who are familiar with graphics. Given that graphics seem to enhance delayed recall, integrating graphical representations into teaching materials could significantly improve learning outcomes. Indeed, teachers already consider graphics as an essential tool for the understanding of scientific subjects (Enzingmüller & Prechtl, 2021; Gheith & Aljaberi, 2015), but their long-term memorization may be equally relevant to education. Still, in our study, we only used very simple graphical representations. Thus, whether our findings hold for more complex (or real-world) data and patterns should be more thoroughly investigated. For complex graphics, we would also expect a significant effect of graphicacy, which was only marginally present here for the immediate recall of elementary numerical information. Note that the participants in the present study all had some training in statistics: a simple graph was surely easily understandable to them. A graphical advantage over text is unlikely to hold for people who are not literate in data visualization.

Future research could also expand the present findings by exploring different types of graphics and their relative effects on memory retention. In fact, the existing literature has mainly focused on how to make more memorable either the graphic itself (Borkin et al., 2013, 2016), its title (Peña et al., 2020), or the general theme conveyed by it (Borgo et al., 2012), rather than directly testing the effects of different data visualizations on the memorization of the content they depict. It is reasonable to speculate that some types of graphics are more suitable for the recall of specific data patterns: for example, a line graph seems particularly informative and efficient when the goal is to notice or to recall an evolution in the data (Ancker et al., 2024), whereas a pie chart might be more adequate if the purpose is to remember the occurrences of a specific level of a categorical variable.

An interesting tangential question is whether graphical representations can be internally elicited from text and tables; in other words, can readers autonomously imagine the information as a graphics and, if so, does it improve their memory encoding of the phenomenon they read about? Evidence suggests that internal images are generated when learning new content, and can be reactivated at recall (Rapp & Kurby, 2008). Whether this is true for graphics as well remains open to empirical investigation. It might be naturally occurring for a minority of students who are highly familiar with graphics, but it would be interesting to see whether explicitly inviting participants to actively imagine the data in a graphical format improves their recall performance.

A last aspect of our study was the inclusion of misleading graphics, where the y-axis was manipulated to amplify or reduce visual trends. Participants exposed to these misleading graphics often overestimated the strength of the recalled trends (both in the delayed- and the immediate-recall tasks), confirming a high susceptibility to visual manipulations of the data (Rho et al., 2023) and thus providing indirect evidence that our behavioral measures of performance did not suffer from a floor effect (i.e., the tasks being too easy for our participants independent of the experimental condition). This aspect of our data highlights the importance of accurate and ethical data visualization practices (Correll, 2019): while graphics can significantly enhance memory retention, they can also enhance the memorization of misleading information.

## Data Availability

All study materials and data are publicly available (https://osf.io/5w2vm/?view_only=832552e639a142fdb9531acef4818525).

## Code availability

All analysis scripts are publicly available (same link as above).
